# The role of outcome expectancies for a training program consisting of meditation, breathing exercises, and cold exposure on the response to endotoxin administration: a proof-of-principle study

**DOI:** 10.1007/s10067-015-3009-8

**Published:** 2015-07-21

**Authors:** Henriët van Middendorp, Matthijs Kox, Peter Pickkers, Andrea W. M. Evers

**Affiliations:** Health, Medical and Neuropsychology Unit, Institute of Psychology, Faculty of Social and Behavioural Sciences, Leiden University, PO Box 9555, 2300 RB Leiden, The Netherlands; Radboud Institute for Molecular Life Sciences, Department of Intensive Care Medicine, Radboud University Medical Center, Nijmegen, The Netherlands; Radboud Institute for Molecular Life Sciences, Department of Anesthesiology, Radboud University Medical Center, Nijmegen, The Netherlands

**Keywords:** Cytokines, Expectancies, Inflammation, Optimism, Proof-of-principle study, Psychology

## Abstract

Expectancies play a major role for the treatment outcome of a broad variety of immune-mediated conditions and may strengthen or mimic the effects of regular long-term therapies. This study adds to a recently published study of Kox et al. (PNAS 111:7379–7384, 2014) on the ability to voluntarily influence the physiological stress response in healthy men after a training program consisting of meditation, breathing techniques, and exposure to cold, which found highly promising results on the clinical, autonomic, and immune response to experimentally induced inflammation (using the experimental human endotoxemia model). Within this project, a number of variables were included to assess the role of generalized (optimism, neuroticism) and specific outcome expectancies (related to the effects of the training on health) on the response to endotoxin administration after training. Indications were found that especially the generalized outcome expectancy optimism is a potential determinant of the autonomic (epinephrine: rho = 0.76, *p* < .01) and immune response (interleukin-10: rho = 0.60, *p* < .05) to induced inflammation after training, whereas more specific expectations with regard to the effects of the training could be especially relevant for the clinical symptom report (flu-like symptoms: rho = −0.71, *p* < .01). This proof-of-principle study provides first indications for potential innovative treatments to change immune-modulating responses by means of psychological mechanisms. If replicated, these findings may be used for predicting training responses and potentiate their effects by means of optimism-inducing interventions in patients with immune-mediated rheumatic conditions.

## Introduction

Psychological factors are supposed to play a major role for the treatment outcome of a broad variety of immune-mediated conditions, such as rheumatoid arthritis [1–3]. In particular, the broad literature on placebo effects has been shown that expectancies about health can induce immune responses that may directly and positively influence health and treatment outcomes, whereas negative expectancies about a treatment can cause an inert substance to create harmful effects or induce unwanted side effects [4–7]. For example, outcome expectancies based on conditioning, such as partial reinforcement of treatment dosages, could lead to a significant reduction in active medication and reported side effects in various conditions, without negatively impacting disease activity and symptom reporting [8–11]. In addition, outcome expectancies based on verbal suggestions on the effectiveness of a treatment have shown important in predicting treatment outcomes. However, particular promising effects have been found for the combination of both (conditioning and verbal suggestions) outcome expectancy mechanisms [8–13]. Thus, outcome expectancies may strengthen or mimic the effects of regular long-term therapies and might be used to find innovative ways to optimize the individual treatment response in conditions requiring long-term pharmacological treatment [6, 14, 15].

A case study of a Dutch individual Wim Hof, who has developed a training program consisting of meditation, breathing techniques, and exposure to cold to voluntarily influence the physiological stress response, revealed that this individual appeared to strongly activate the sympathetic nervous system and attenuate the immune response in reaction to controlled experimentally induced inflammation (using the experimental human endotoxemia model) [16]. Recently, a proof-of-principle randomized controlled trial (RCT) in 12 healthy male volunteers compared to 12 non-trained volunteers examined the effects of this training program in others, showing remarkably similar effects [17]. If replicated in a larger sample, such a training program could have important implications for the treatment of, for instance, autoimmune diseases.

In order to gain insight into potential variables that may predict the effectiveness of such training, it is relevant to know whether specific psychological variables, such as outcome expectancies, are related to the response to the experimental inflammation after training. More stable individual differences (e.g. personality characteristics of optimism or neuroticism) in expectancies can be distinguished from more specific and situation-depending expectancies regarding the outcome of a particular treatment or intervention (states of specific outcome expectancies). The personality characteristic of optimism (the general expectation that good things will happen) and neuroticism (the tendency to experience negative emotional states and to view the world as threatening) represents generalized (positive versus negative) outcome expectancy characteristics, respectively. Both have shown to predict future outcomes of morbidity and even mortality in various populations and have been related to stress system and immune system functioning [18–20]. Outcome expectancies that are more specific, situation-depending, and directly focus on the outcomes of a certain treatment have also shown to affect the stress and immune systems, next to effects on more clinical symptom reports [6,20]. As outcome expectancies are assumed to change the psychophysiological stress response, the role of outcome expectancies may be especially visible and relevant in the response to a stressor, such as induced inflammation [13].

The current study explores the role of generalized (optimism, neuroticism) versus specific (directed to the training effects) outcome expectancies in the clinical, autonomic, and immune response elicited by endotoxin administration after the previously described training program.

## Materials and methods

### Design

This study was performed as part of a proof-of-principle randomized controlled trial in 24 healthy Dutch male volunteers. The study protocol was approved by the local ethics committee of the Radboud University Medical Center (CMO2012/454). All subjects provided written informed consent, and the study was conducted in accordance with the Declaration of Helsinki and Good Clinical Practice guidelines. As reported in more detail in the paper previously published on the effects of the training on the response to endotoxin administration [17], subjects were screened and then randomly allocated to the trained group (*n* = 18, of whom 12 were randomly assigned to the endotoxemia experiment day) or the control group (*n* = 12). Participants filled out questionnaires on generalized outcome expectancies before training and questions on specific outcome expectancies before and after training and at the end of the endotoxemia test day. The current study reports on the results of the trained group.

### Training program

The trained group was trained by four trainers. The 4-day group program that took place in Poland consisted of three main elements: 3^rd^ eye meditation including visualizations aimed at total relaxation, exposure to cold (e.g., dipping in ice-cold water or hiking up a snowy mountain bare-chested), and breathing techniques (hyperventilation followed by breath retention and deep inhalations and exhalations). After the 4-day group program, the subjects practiced the techniques they learned daily by themselves at home (2–3 hours a day) until the endotoxemia experiment day (5–9 days later). A final group training took place, and at the end of this day, 12 trained subjects were randomly selected for participation in the endotoxemia experiments in order to allow for subject replacement in case of an adverse event or illness.

### Experimental human endotoxemia model

The endotoxemia experiment took place at the intensive care department of the Radboudumc. The procedures on the endotoxemia experiment day are described elsewhere [17]. In short, purified lipopolysaccharide (LPS, US Standard Reference Endotoxin *Escherichia coli* O:113) solution was administered as an i.v. bolus injection at a dose of 2 ng/kg body weight in 1 min at T0. From 30 min before LPS administration to 2.5 hours after administration, trained participants practiced the breathing techniques (hyper/hypoventilation cycles for the first 1.5 hours and deep inhalation and exhalation in combination with tightening muscles for the next hour).

### Measures

#### Outcome expectancies

##### Generalized outcome expectancies

The Life Orientation Test-Revised [21] was used to measure optimism by means of six items (and four filler items) on a 5-point Likert scale varying from “strongly disagree” to “strongly agree.” An example item is “In uncertain times, I usually expect the best.” The neuroticism scale of the Big Five Inventory [22] was used to measure neuroticism by means of eight items on a 5-point Likert scale varying from “disagree strongly” to “agree strongly.” An example item is “I see myself as someone who worries a lot.”

##### Specific outcome expectancies

Four items were constructed on expectancies regarding the effects of the training on the response to endotoxin administration and on general health, to be answered on a 10-point numeric rating scale varying from “no influence” to “very much influence.” The items were “Please indicate how much influence you think that the training has on… 1) the functioning of your immune system, 2) your physical complaints (e.g., experiences of pain and fatigue), 3) the degree to which you will experience physical complaints after the administration of endotoxin, and 4) the degree to which you will become sick from the administration of endotoxin”. The internal consistency (Cronbach’s alpha) of the items was .85.

##### Response to endotoxin administration

In line with the main study [17], the specific time point with the strongest clinical, autonomic, or immune response to endotoxin administration was used as outcome measurement.

##### Clinical symptom report

LPS-induced flu-like symptoms (headache, nausea, shivering, muscle, and back pain) at 90 min after endotoxin administration (T90) were scored on a 6-point Likert scale varying from no symptoms to worst ever experienced.

##### Autonomic response

Details of the blood sampling and collection is reported elsewhere [17]. Plasma epinephrine concentrations at the time of endotoxin administration (T0) were measured using routine analysis methods also used for patient samples (HPLCy with fluorometric detection).

##### Immune response

Concentrations of the anti-inflammatory cytokine interleukin (IL)-10 at 60 minutes after endotoxin administration (T60), the pro-inflammatory cytokine tumor necrosis factor alpha (TNF-α) at 90 minutes (T90), and IL-6 and IL-8 at 120 minutes after endotoxin administration (T120) were measured using a simultaneous Luminex assay according to the manufacturer’s instructions (Milliplex; Millipore) [17].

### Statistical analysis

As we were interested specifically in the effects of outcome expectancies on the response to endotoxin administration after training and as specific outcome expectancies regarding the effects of the training could only meaningfully be assessed in the trained group (*n* = 12), analyses were performed in the trained group that underwent the endotoxin administration only. Variables were screened for deviations of normality. Changes in specific outcome expectancies from before the training to after the experiment were explored by means of analysis of variance. Spearman’s rank correlations between outcome expectancies and the response to endotoxin administration were explored.

## Results

### Clinical, autonomic, and immune response elicited by endotoxin administration

As reported in detail elsewhere [17], the trained group (*n* = 12) reported fewer flu-like symptoms; showed profoundly increased plasma epinephrine levels at the start of the experiment, followed by a more rapid and profound increase in anti-inflammatory IL-10 levels; and lower subsequent pro-inflammatory TNF-α, IL-6, and IL-8 levels than the non-trained group in response to endotoxin administration.

### Outcome expectancies

As expected for healthy young males, participants in the trained group were overall rather optimistic (*M* ± SD = 18.00 ± 3.46, range 12–24) and low in neuroticism (*M* ± SD = 2.11 ± 0.55, range 1.13–3.13), with both variables showing normal distributions (skewness and kurtosis <.62). With regard to specific outcome expectancies, participants showed an increase in the expected effects of the training on their response to endotoxin administration and health from before the training to after the endotoxemia experiment (before training: *M* ± SD = 27.69 ± 6.16, before endotoxemia experiment: *M* ± SD = 31.68 ± 3.11, after endotoxemia experiment: *M* ± SD = 34.28 ± 1.71; *F*_2,22_ = −10.56, *p* = .003).

### Associations between outcome expectancies and responses to endotoxin administration

Spearman’s rank correlations of generalized and specific outcome expectancies with the clinical, autonomic, and immune response elicited by endotoxin administration are reported in Table 1.

**Table 1:** Spearman’s rank correlations of generalized and specific outcome expectancies with the clinical, autonomic, and immune response elicited by endotoxin administration after the training program in healthy young men (*n* = 12)

Response to endotoxin administration	Clinical	Autonomic	Immune			
Outcome expectancies	Flu-like symptoms (T90)	Epinephrine (T0)	IL-10 (T60)	TNF-α (T90)	IL-6 (T120)	IL-8 (T120)
Generalized outcome expectancies						
Optimism (LOT-R)	−.38	.76**	.60*	−.32	−.26	−.32
Neuroticism (BFI)	.10	−.32	−.09	.27	.22	.11
Specific outcome expectancies	−.71**	.28	.33	.00	.16	−.15

*IL* interleukin, *TNF* tumor necrosis factor, *LOT-R* Life Orientation Test-Revised, *BFI* Big Five Inventory

*p < .05; **p < .01

A higher degree of optimism was associated with higher plasma epinephrine levels (Rho = 0.76, *p* < .01) and IL-10 levels (Rho = 0.60, *p* < .05). Neuroticism was not significantly associated with the response to endotoxin administration. A more positive expectation of the effects of the training was associated with a lower flu-like clinical symptom report (Rho = −0.71, *p* < .01).

## Discussion

This proof-of-principle study in a relatively small sample of healthy young men provides first indications that the generalized outcome expectancy optimism is a potential determinant of the autonomic and immune response to intravenous administration of bacterial endotoxin after a short-term training program consisting of meditation, breathing exercises, and cold exposure. More specific outcome expectancies related to the training’s effects may be especially relevant for the clinical symptom report.

The results found for generalized outcome expectancies of optimism are in line with previous studies demonstrating an association between optimism and both stress and immune system functioning e.g., [18–20]. However, this it is to our knowledge the first study to suggest that optimism contributes to an increased autonomic and repressed evoked immune response in a standardized and reproducible experimental inflammation model. Also, the finding that specific outcome expectancies are especially related to clinical symptom report (e.g., pain experience) corresponds with previous research [6, 20]. That generalized and specific outcome expectancies regarding the effects of the training were not associated with similar aspects of the response to endotoxin administration is in line with a previous study that showed that specific expectancies regarding the level of experienced pain in response to cold pressor did not mediate or moderate the association between optimism and pain experience [20]. Thus, generalized and specific expectancies appear to independently impact clinical and immune responses that are also relevant for rheumatic diseases. Future research into the mechanisms responsible for the effects of generalized and specific expectancies on clinical, autonomic, and immune responses is warranted. However, the results of this study should be interpreted with caution and need to be replicated in larger samples in order to show its stability and generalizability. Also, examining these associations in samples showing disturbed autonomic and immune responses as part of the disease process, such as in chronic inflammatory rheumatic conditions, would show their potential clinical relevance. The findings suggest a possible role of especially optimism as a predictor of the autonomic and immune response to inflammatory stress after a brief training program. If replicated, these findings may be used for predicting training responses and potentiate their effects by means of optimism-inducing interventions e.g., [20,23,24] in patients with immune-mediated rheumatic conditions.
